# The Roles of Anxiety and Self-Esteem in the Risk of Eating Disorders and Compulsive Buying Behavior

**DOI:** 10.3390/ijerph192316245

**Published:** 2022-12-04

**Authors:** Concetta De Pasquale, Martina Morando, Silvia Platania, Federica Sciacca, Zira Hichy, Santo Di Nuovo, Maria Catena Quattropani

**Affiliations:** 1 Section Philosophy and Social Sciences, Department of Educational Sciences, University of Catania, 95124 Catania, Italy; 2 Section Psychology, Department of Educational Sciences, University of Catania, 95124 Catania, Italy

**Keywords:** compulsive buying behavior, mediation, self-esteem, eating disorder, trait anxiety, multigroup

## Abstract

In contemporary society, following the sudden changes that occur, different forms of addiction are becoming popular. Of note are the new addictions and concepts of poly-dependencies that involve common behaviors and trap people who suffering from them in a vicious circle. The main goal of this study is to investigate the possible mediating role that self-esteem had between trait anxiety and two specific new addictions: compulsive buying behavior and eating disorders. Furthermore, it was verified through a multigroup analysis that trait anxiety had a greater effect on eating disorders in a group of compulsive consumers. Three-hundred and fifty-two participants (67.9% women) were enrolled to participate in this study. The results showed that there was a direct effect of trait anxiety on eating disorders and on compulsive buying behavior; self-esteem mediated the effect of trait anxiety on eating disorders and compulsive buying behavior with specific differences; multi-group analysis showed differences in anxiety’s effect on eating disorders between compulsive and non-compulsive consumers; the group of compulsive consumers revealed a significant and stronger effect of trait anxiety on eating disorders in all domains identified. Further research is recommended to better understand the predictors of these disorders and to contribute to a more effective preventive intervention.

## 1. Introduction

The concept of polydependence, and the more general concept of addiction, represent a real social emergency [1,2]. The fifth edition of the Diagnostic and Statistical Manual of Mental Disorders (DSM-5) included changes in the classification of ‘Substance Related Disorders and Dependence’ in the direction of increasing the recognition of addiction behavior without substances [3]. Some conditions such as compulsive buying, kleptomania, compulsive sexual behavior, show neurobiological phenomena parallel to substance use disorders [4,5]. However, several addictions have not been included in current classifications yet; for example, compulsive buying is commonly classified as a behavioral addiction, although the nosographic diagnosis is not included in official classifications and diagnostic manuals yet [6,7,8,9,10,11,12,13]. Substantial research revealed prevalence estimates of compulsive buying between 6% and 7% and indicates that young people are more prone to develop this behavior. Furthermore, European data suggest an increase in compulsive buying in the adult population [14]. Compulsivity can easily emerge as a personality trait in people with an uncontrollable desire to shop. These people also present lower self-esteem, than normal consumers [15].

The concept of addiction has expanded in recent years by introducing “new addictions” including compulsive behavior. These obsessive and compulsive behaviors cause unhealthy eating patterns that tend to develop eating disorders. People affected by these disturbed eating habits typically have an unhealthy body image, caused by poor opinions of themselves, and co-occurring disorders such as depression and trait anxiety, many turn to substance abuse to cope [16,17,18].

## 2. The Relationship between Compulsive Buying and Eating Disorder Symptoms

Compulsive buying is a psychopathological pattern of intrusive, recurring thoughts and impulses oriented towards the search for and purchase of goods that are often superfluous or have an economic value beyond the individual’s means. People often recognize the item as non-essential, unnecessary or excessively expensive, but are unable to resist the impulse to purchase; these people are described as “overly preoccupied with shopping and driven by an uncontrolled shopping motivation that drives them to invest so much time and resources that important areas of life are compromised” [19].

Compulsive shopping has high rates of comorbidity with other disorders, including eating disorders [20,21,22].

Eating disorders are defined as an alteration in eating behavior with excessive preoccupation with body weight that compromises physical health or psychosocial functioning. Anorexia nervosa (AN) involves a refusal to maintain a normal weight, fear of gaining weight, and a disturbed perception of body shape, whereas bulimia nervosa (BN) is characterized by recurrent and/or uncontrollable binge eating with inadequate compensatory behavior to prevent weight gain and excessive preoccupation with body shape. Binge-eating disorder (BED), included in DSM-5 as a diagnostic category, is characterized by recurrent episodes of binge eating in the absence of regular compensatory behaviors such as vomiting or laxative abuse [3].

Eating disorders occur commonly in the general population and prevalence estimates worldwide are anorexia nervosa 0.21% (95% confidence interval (CI), 0.11–0.38), bulimia nervosa 0.81% (95% CI, 0.59–1.09) and binge-eating disorder 2.22% (95% CI, 1.78–2.76) with a higher prevalence in females than males. They also have high rates of psychiatric comorbidity, particularly anxiety disorders [23].

The literature over time has proposed several studies investigating the relationship between compulsive-buying behavior (CB) and eating disorders.

Several studies [6,24,25,26] reported that the prevalence rate of eating disorder symptoms in CB is 36.9 per cent. Other studies, such as that of Christenson et al. [27] comparing individuals with CB with a control sample, found that individuals with CB have a higher lifetime prevalence of developing anxiety, substance use and eating disorders. Additionally, the contribution of Black et al. [28] also showed that individuals with CB were more likely to suffer from anorexia nervosa (12.1% vs. 4.6%) and bulimia nervosa (9.1% vs. 0%) compared to a control sample.

Lejoyeux et al. [29] also found in a sample of hospitalized depressed patients a 31% proportion of patients who were also diagnosed with CB. In addition, according to the same study, depressed patients with CB more often suffered from recurrent depressive disorder, kleptomania, bulimia and benzodiazepine/addiction disorder.

In another study, Fernandez-Aranda et al. [30] found a high prevalence (11.8%) of CB disorder in a sample of patients with various eating disorders. This study also revealed that impulse control disorders, including compulsive buying, were more frequent in patients with binge-eating symptoms and had a higher correlation with harm avoidance, neuroticism, cognitive impulsivity and lower self-directedness.

Due to the high comorbidity between CB and symptoms related to uncontrolled-eating disorders, it is possible that similar temperamental characteristics underlie both CB and eating disorder-related symptoms [31].

## 3. The Effect of the Trait Anxiety

People affected by trait anxiety have the tendency to have negative thoughts about future events that have not happened yet but have the possibilities to have negative outcomes [32]. This experience can affect a variety of situations across time, including eating behaviors, compulsive buying and addictions in general.

Indeed, there are several studies in the literature that have specifically examined the role of trait anxiety in compulsive buying [33,34,35,36,37]. These studies revealed the comorbidity between trait anxiety and compulsive buying, confirming previous evidence of the comorbidity of various behavioral addictions, such as pathological gambling and internet addiction, with trait anxiety, personality disorders and mood disorders.

Furthermore, factors frequently associated with eating disorders appear to be high levels of negative effects and, in particular, a marked difficulty in managing trait anxiety [38,39,40,41]. Indeed, several studies have confirmed that there is a prevalence of anxiety disorders for all eating behaviors and eating disorders compared to the general population. Furthermore, it was found that higher levels of trait anxiety are more often associated with a significant increase in compensatory behaviors, body dissatisfaction and binge eating [41,42,43].

It has been found that trait anxiety, social comparison and personality factors such as conscientiousness and perfectionism are closely related to eating disorders [44,45,46,47,48], while low self-esteem, high levels of trait anxiety symptoms and depression, obsessive and impulsive behavior have been correlated with compulsive shopping behavior [49,50,51].

## 4. The Potential Mediation Role of the Self-Esteem

Self-esteem could be considered a significant indicator of health and well-being. Over time, the literature has highlighted the importance of the relationship between self-esteem, mental well-being and eating behavior [52,53]. In this sense, self-esteem seems to have effects on psychological and social adaptation and in the prevention of risk behavior [54]. Other studies identified that low self-esteem, specific personality traits and emotional experiences appear to be risk factors for eating disorders [55]. Particularly among younger people, self-esteem plays an important role in the construction of personal body image but is also a mediating factor for eating disorders. Indeed, low self-esteem often corresponds with the presence of eating disorders, the symptoms of which further exacerbate the symptoms associated with low self-esteem, creating a very dangerous and constant vicious circle [56,57].

It is very important to analyze the relationship between self-esteem, weight loss behavioral patterns, with the risk of developing eating disorders and to investigate the relationships of these factors among individuals most vulnerable to developing eating disorders. The study by Cella et al. [58] showed the relationship between self-esteem, body disinvestment and binge eating in adolescence. De Pasquale et al. [59] highlighted the relationship between the drive for thinness, conscientiousness and bulimic traits during the first and second years of adolescence, emphasizing the importance of a preventive assessment of suggestive psychological traits.

The role of self-esteem has also been examined in connection with compulsive buying. In a study by Biolcalti [60], for example, it was reported that self-esteem was a strong predictor of compulsive buying for both genders, whereas fear of negative evaluation seemed to play a mediating role between self-esteem and compulsive buying behavior only for women. Unfortunately, the literature does not present many studies on this specific topic, but there is a real opportunity for researchers to develop new literature that can provide information.

### Hypothesis

On the basis of what has been analyzed so far, it has been hypothesized for the present research that:

**H1a–c.**  *There is a direct effect of trait anxiety on the risk of eating disorders and on compulsive-buying behavior.*

**H2a–c.**  *There is an indirect effect (mediation) of self-esteem on the relationship between trait anxiety and the risk of eating disorders and compulsive-buying behavior.*

Additionally:

**H3a.**  *Trait anxiety as a risk factor in compulsive consumers, dissimilar in non-compulsive consumers.*

**H3b.**  *(If H3a is supported) trait anxiety as predictive factor of eating disorders in compulsive consumers.*

## 5. Materials and Methods

### 5.1. Participants and Procedure

Data were collected through an online survey using the Google Form platform shared on social media groups (Facebook, Telegram, WhatsApp). An individual and anonymous questionnaire with several scales was used. Respondents were presented with a participant information sheet and an informed consent form, which only once accepted, led to the survey with instructions on how to complete it.

Data collection was carried out from October 2020 to February 2021. It was used convenience sampling, enrolled from the general population. The participants were healthy volunteers.

Three hundred and fifty-two participants (67.9% women) were enrolled to participate in this study, aged between 18 and 37 (*M* = 24.5, *SD* = 3.4), 49.7% of them were undergraduate. As for educational level, 46.1% of the sample completed 13 years of schooling, while the remaining 53.9% completed a minimum of 16 years of schooling. Most of the participants (54%) were students; the income variable (68%) declared that they belonged to the range from 25,000 EUR to 35,000 EUR per year.

The study was carried out in accordance with the Declaration of Helsinki, and the protocol was authorized by the Internal Ethics Review Board of the Department of Educational Sciences (Section of Psychology) of the University of Catania; the research procedures followed all the indications provided by the guidelines of the AIP (Italian Association of Psychology) and its Ethical Council.

### 5.2. Measures

#### 5.2.1. Eating Disorder Inventory (EDI-2)

The EDI is considered the gold standard for the assessment of eating disorder psychopathology [61]. The EDI-2 [62] Italian adaptation by Rizzardi, Trombini and Trombini [63], is a self-report questionnaire consisting of 91 items, scored on a 6-point Likert-type scale (from 1 = never to 6 = always), where higher scores are suggestive of the presence of disordered eating patterns. The Italian version of the instrument showed a good internal consistency (Cronbach’s alpha between 0.78 and 0.84 in clinical population), a good construct validity and a good discriminant validity for screening clinical and non-clinical populations (Cronbach’s alpha from 0.78 to 0.88) [33]. Cronbach’s alpha of the EDI-2 in the current study was 0.92. In particular, EDI-2 consisted of 8 primary subscales (64 items) and 3 additional subscales (27 items), investigating the following dimensions: drive for thinness (α = 0.88), bulimia (α = 0.85), body dissatisfaction (α = 0.88), ineffectiveness (α = 0.89), perfectionism (α = 0.83), inter- personal distrust (α = 0.97), interoceptive awareness (α = 0.95), maturity fears (α = 0.92), asceticism (α = 0.95), impulse regulation (α = 0.93), and social insecurity (α = 0.95).

#### 5.2.2. Binge Eating Scale (BES)

The Binge Eating Scale (BES) developed by Gormally, Black, Daston and Rardin [64], with the Italian version by Di Bernando et al. [65] and Imperatori et al. [66]. The BES is a 16-item instrument designed to measure binge eating symptomatology. It evaluates the main behaviors, sensations and cognitive aspects associated with bulimic episodes (e.g., eating quickly or a large amount of food consumed; feeling of loss of control or inability to stop eating, guilty). It is asked to choose from the statements that best describe their mood or habitual behavior. Total scores are obtained with sum and range from 0 to 46, with higher scores indicating greater severity. Specifically, Marcus et al. [67] identified clinical cut-off scores: <17 total score representing none-to-minimal risk, range 18–26 representing moderate severity, and total score >27 representing severe binge eating problems. In this study, the internal consistency, calculated using Cronbach’s alpha, was 0.89.

#### 5.2.3. Rosenberg Self-Esteem Scale (RSE)

The RSES is an instrument developed by Rosenberg [68], whose Italian version was edited by Prezza et al. [69]. It is a self-report instrument composed of 10 items, investigating the degree of global and personal self-esteem, measured on a 4-point Likert scale (from “strongly agree” to “strongly disagree”). The Italian version of the scale revealed the presence of two correlated factors assessing self-derogation and self-recognition.

Scores ranged between 0–30. The score below the cut-off (15 points) indicated low levels of self-esteem and a low sense of efficacy. Cronbach’s alpha of this instrument in the current study was 0.79 for second order factor, while was (α = 0.76) for self-derogation and (α = 0.82) for self-recognition.

#### 5.2.4. Compulsive Buying Scale (CBS)

The Compulsive Buying Scale, conceptualized by Faber and O’Guinn in 1992 [70] (Italian adaptation by Tommasi and Busonera [71]), is a seven-item clinical screener to identify compulsive buyers within the general population. The above-mentioned scale is measured with a 5-point Likert scale (from “completely agree” to “completely disagree” and from “very often” to “never”). The scores were calculated with a specific equation and scored less than −1.34 identified an individual as a compulsive buyer. For this specific sample, Cronbach’s alpha was 0.76.

#### 5.2.5. The State-Trait Anxiety Inventory (STAI)

The State-Trait Anxiety Inventory (STAI) developed by Spielberger et al. [72] is one of the most commonly used self-report measures of anxiety. Form Y (Italian version) used in this study, which is the most popular version, had 20. All items are rated on a 4-point scale (e.g., from “Almost Never” to “Almost Always”). Higher scores indicate greater anxiety. The trait scale has 7 reversed scores items. The Cronbach’s alpha for our specific sample was 0.89 [73].

### 5.3. Statistical Analysis

SPSS (version 27.0 for Windows; IBM Corp., Armonk, NY, USA) was used for the descriptive and correlations analysis of the variables in this study. Furthermore, a two-sample *t*-test is used to test gender differences. Normality of data in each analysis (*p* > 0.05) was evaluated by Shapiro–Wilk tests for normality, for each variable in the total sample and for each gender.

To test the hypotheses H1 and H2, a structural equation modeling (SEM) was used. Tests were completed in AMOS 25.0 [74]. To investigate the hypotheses H3a and H3b, invariance tests and multigroup analysis were carried out.

To establish the best factor model to fit the data, a series of Confirmatory Factorial Analyses (CFA) were carried out on the dataset. Harman’s single-factor test [75] is used to examine the problem of common-method variance (CMV). This analysis is used to check whether variance in the data can be largely attributed to a single factor. The models’ goodness of fit was evaluated through several indexes. Specifically, it was used the Comparative Fit Index (CFI), the Goodness-of-fit statistic (GFI), the Standardized Root Mean Square Residual (SRMR), the Root Mean Square Error of Approximation (RMSEA), and the Tucker–Lewis Index (TLI).

Traditionally, an omnibus cut-off point of 0.90 has been recommended for the GFI index [76]. RMSEA in the range of 0.05 to 0.10 was considered an indication of fair fit and values above 0.10 indicated marginal fit [77]. It was then thought that an RMSEA of between 0.08 to 0.10 provides a poor fit and below 0.08 shows a good fit [77]. However, more recently, a cut-off value close to 0.06 [78] or a stringent upper limit of 0.07 [79] seems to be the general consensus. Values for CFI range between 0 and 1 with Bentler and Bonnet [80] recommending values greater than 0.90 indicating a good fit. More recent suggestions state that the cut-off criteria should be TLI ≥ 0.95 [78]. A cut-off criterion of CFI ≥ 0.90 was initially proposed; however, other studies have shown that a value of CFI ≥ 0.95 is presently recognized as indicative of good fit [78,81]. Values for the SRMR range from zero to 1.0 with well-fitting models obtaining values less than 0.05 [82,83]. An SRMR of 0 indicates perfect fit.

Furthermore, χ^2^ values and Δχ^2^ values between the competing models were presented, but they are being sensitive to sample size [84], it was advanced with Akaike Information Criterion (AIC) and Bayesian Information Criterion (BIC) (lower values indicate better fit).

To test the indirect correlation of self-esteem in the relationship between trait anxiety and eating disorders and compulsive buying behavior (H2), mediation analysis was performed by the structural equation model. Following the recommendations of James and colleagues [85] and of Shrout and Bolger [86] on expected proximal and distal effects, two regression models were simultaneously applied assuming that the total effect of the dependent variable on the independent variable is different from the direct effect of the variable. The indirect effect was tested using a bootstrap estimation approach on 2000 samples and a percentile method corrected for 95% bias [87].

To evaluate invariance tests and multigroup analysis, two groups were extracted from the original sample: compulsive buyers (50, 35.5%) and non-compulsive buyers (91, 64.5%). Afterward, the maximum likelihood (ML) estimation method was used to adjust the model individually to each group eliminating the items that did not contribute to the adjustment quality and then tested for model estimation across the groups [88,89]. Data analysis involved three different steps: testing measurement model invariance; testing structural model invariance; testing structural path coefficients differences.

The invariance of the measurement model across the two multigroup was tested by comparing the unconstrained model (i.e., with all parameters free) to the model with measurement weights constrained (i.e., the measurement model per se). In this second step of this data analysis, the invariance of the structural model was tested across the two multigroup, considering correct the model with measurements weights constrained and comparing the model with the unconstrained structural coefficients to the constrained model (i.e., with structural weights constrained). According to the results of the multigroup invariance, the differences in structural path coefficients were investigated, that is, if there was a significant effect of trait anxiety on eating disorders among compulsive and non-compulsive consumers.

## 6. Results

As the first step, an analysis of gender differences according to the current literature was carried out [90,91]. The results claimed that females, on average, had higher compulsive buying scores, [t (350) = −2.71, *p* < 0.05]. Furthermore, females reported higher scores of body dissatisfaction [t (350) = −2.86, *p* < 0.05]. On the contrary, males reported greater level of perfectionism [t (350) = 3.14, *p* < 0.05]. Gender differences for trait anxiety levels were not revealed (*p* > 0.05).

### 6.1. Descriptive Statistics of the Scales, Alpha and Correlations

Descriptive statistics of the scales (means and standard deviations), Cronbach’s alphas and intercorrelations among the variables were shown in Table 1. The correlation matrix showed that there was a positive correlation between eating disorder and binge eating (r = 0.49, *p* < 0.001), trait anxiety (r = 0.45, *p* < 0.001) and compulsive buying (r = 0.34, *p* < 0.001). On the contrary, there was a negative correlation between eating disorder and self-esteem (r = −0.19, *p* < 0.001).

### 6.2. Confirmative Factorial Analysis of the Measures

All the previous variables were measured from the same source, and therefore common-method bias may occur. A CFA according to Harman’s single-factor test to examine where common-method variance was a problem was carried out. The comparison between the hypothesized model and a model with one factor (with all items loading on a unique factor) revealed that the former provided a better fit for the data in all the CFA fit measures (e.g., 6-factor model: χ^2^ (49) = 133.70, *p* < 0.001, CFI = 0.89, GFI = 0.90, SRMR = 0.04, RMSEA = 0.04 (0.061 − 0.049), AIC = 117.689, BIC = 205.371; 1-factor model: χ^2^ (52) = 235.25, *p* < 0.001, CFI = 0.66, GFI = 0.68, SRMR = not possible to estimate, RMSEA = 0.19, and AIC = 3874.15). The differences were significant according to a comparison of the models’ χ^2^ values and degrees of freedom: Δχ^2^ (3) = 101.55 (*p* < 0.001). No evidence for common-method bias in the data was found.

### 6.3. Structural Model

**Figure 1:**
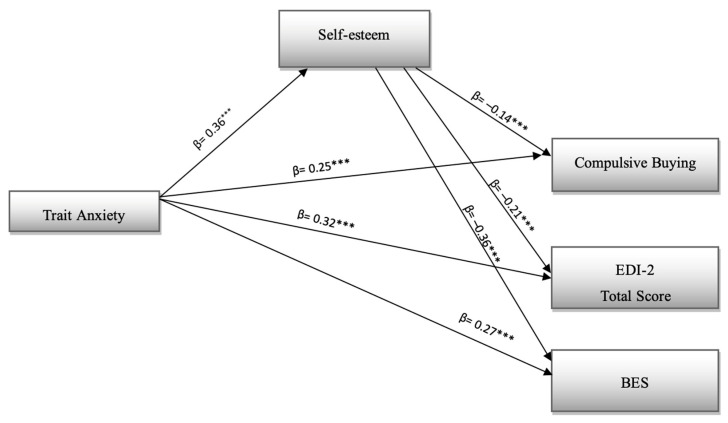
Mediation analysis. *** *p* < 0.001.

The model in Figure 1 reported a mediation analysis conducted using AMOS software, version 27.0 [74,92].

Compulsive Buying, total score of EDI-2 and Binge Eating Scale (BES) were entered as dependent variables and Trait Anxiety as an independent variable; moreover, Self-esteem was entered as a mediator.

All variables were entered as latent variables, except for outcomes that were entered as observed variables. The bootstrapping method (2000 samples) was used with bias-corrected (BC) confidence intervals to obtain more powerful confidence interval limits for indirect effects (95% CI) [93].

### 6.4. Direct Effects

The results shown in Figure 1, revealed that there were positive and direct effects for the path Trait Anxiety–Compulsive Buying (H1a, β = 0.25, *p* < 0.001); for the path Trait Anxiety–EDI 2 (H1b, β = 0.32, *p* < 0.001), for the path Trait Anxiety–BES (H1c, β = 0.27, *p* < 0.001), and for the path Trait Anxiety–Self Esteem (β = 0.36, *p* < 0.001). Results prove that hypothesis 1 is almost totally confirmed by the results. There were also negative and direct effects between the mediator and the outcomes: path Self Esteem–Compulsive Buying (β = −0.14, *p* < 0.001); path Self Esteem–EDI 2 (β = −0.21, *p* < 0.001); path Self Esteem–BES (β =−0.36, *p* < 0.001).

### 6.5. Standardized Indirect Effects

As showed in Table 2, Self-esteem partially mediated the relationship between Trait Anxiety and BES (H2a), Trait Anxiety and EDI-2 (H2b) and Trait Anxiety and Compulsive Buying (H2c). Thus, hypothesis 2 is totally confirmed.

### 6.6. Multigroup Analysis the Effect of Trait Anxiety on Eating Disorders between Compulsive and Non-Compulsive Consumers

To test hypothesis H3a, a multi-group analysis was carried out to explore differences on the effect of trait anxiety on eating disorders between compulsive and non-compulsive consumers; the selection of the sample was made considering the balance of two main variables: the cut-off threshold of compulsive-buying behavior and the gender variable. Therefore, a subgroup of 141 participants (compulsive 50, 35.5%; non-compulsive 91, 64.5%; 63 men, 44.7%, 78 women, 55.3%) was selected from the original sample. The invariance of the structural model was tested across the three multigroup, considering correct the model with measurement weights constrained and comparing the model with the unconstrained structural coefficients to the constrained model (i.e., with structural weights constrained). As shown in Table 3, the structural models with constrained coefficients presented a significant worse adjustment to groups compared to the model with free coefficients because *p*Δχ^2^ < 0.001.

These results proved that the causal model, concerning the effect of trait anxiety on eating disorders, was not invariant among groups of compulsive and non-compulsive consumers, and their interaction. Therefore, the null hypothesis was rejected for all hypotheses, supporting the differences in the structural models.

### 6.7. Testing Structural Path Coefficients Differences

Due to the results of the multigroup invariance, it was investigated whether there was a significant effect of trait anxiety on eating disorders among compulsive and non-compulsive consumers. The findings confirmed hypothesis H3b, showing in the group of compulsive consumers a significant and strong effect of trait anxiety on eating disorders in all domains identified (see Table 4). It also emerged Anxiety Trait on Drive for thinness (β = −0.39, *p* < 0.001), Anxiety Trait on Bulimia (β = 0.36, *p* < 0. 001), Anxiety Trait on Body Dissatisfaction (β = 0.27, *p* < 0.001), Anxiety Trait on Maturity Fears (β = 0.21, *p* < 0.05) and Anxiety Trait on Ineffectiveness (β = 0.26, *p* < 0.05). These results confirmed data and findings in the current literature: trait anxiety is a very common issue and could predict many different addictions [94]. Specifically, the role of self-esteem in relation to compulsive buying behavior, as indicated by Faber and O’Guinn [70,95], and the role of BES in identifying dysfunctional behaviors towards food is supported in both clinical [66] and non-clinical populations [96].

## 7. Discussion

The main objective of this study was to investigate the possible mediating role that self-esteem had between trait anxiety and two specific new addictions: compulsive buying behavior (CB) and eating disorders. Furthermore, it was investigated whether trait anxiety was a predictive factor of eating disorders in compulsive consumers.

The results of our study are partly consistent with the international literature, as in previous studies, our female sample reported higher compulsive buying scores than men [36,37]. Additionally, the present sample supported the tendency of individuals with CB to use consumption as a means of escape and regulation of negative feelings (such as anxiety) [35,97].

Consistent with the extensive literature, the study confirmed the high levels of body dissatisfaction in the female sample compared to the male sample [23,98,99,100,101,102], such as in studies by Dittmar et al. [103] and Mueller et al. [104]. Females, in contrast with males, present higher CB scores; this demonstrates how levels of compulsive buying behavior is not related to gender but to other factors such as materialism or socio-cultural context [90,91,105,106]. Other more recent studies [107], reported that perfectionism only predicted binge eating symptoms among adolescent boys, suggesting that males experience a different set of concomitants to these symptoms than females; therefore, this explains how the current culture’s obsession with beauty and physical attractiveness is not a gender-related aspect nowadays.

Several studies and meta-analyses tried to identify predisposing and predictive factors investigating the risk factors associated with eating disorders. Besides biological and social factors, internalization of the ideal of thinness, perfectionism, negative feelings, preoccupation with weight, and overestimation of appearance were identified as psychological risk factors [107,108]. At the same time, in addition to these considerations, low self-esteem, anxiety, depression and emotional eating were often discussed in connection with eating disorders and appear to be important components of the symptomatology.

The model proposed in our study fully supported the findings promoted in the literature, which have found elevated trait anxiety among those suffering from eating disorders, e.g., [109,110,111]. It would therefore appear plausible to deduce that trait anxiety may also be a precursor to the development of eating disorders in this sample [112]. Furthermore, our findings confirmed and supported the existing evidence of an association between compulsive buying and trait anxiety [113]. CB is often associated with other behavioral addictions [112], such as mood disorders, anxiety disorders, substance use, other impulse-control disorders and eating disorders [30]. Thus, based on these results, hypotheses H1a–c are completely confirmed.

The relation between these constructs, which is also valid for the examined sample, could be understood by interpreting compulsive consumer behavior as a means through which to achieve immediate, albeit short-term relief from the anxiety, low self-esteem or depression they are experiencing [114]. Consequently, compulsive buying reflects a way of coping with life challenges caused by low self-esteem and self-image concerns [115], as well as emotional tensions.

Based on the considerations that low levels of self-esteem significantly impact eating disorders [58,116] and levels of compulsive buying [29,55], one of the aims of our study was to investigate how self-esteem could mediate the relationship between anxiety, eating disorders and compulsive buying behavior. The data showed that self-esteem partially mediated the relationship between trait anxiety and BES, trait anxiety and EDI-2 and trait anxiety and compulsive buying behavior. This finding, therefore, supported our conclusion and confirmed the hypotheses formulated (H2a–c) of the important role played by self-esteem, in the relationship between anxiety and behavioral addictions, which is able to reduce the effect of the former on the other variables. This result leads to the possibility of investing and working both in prevention and intervention on the population groups identified, since it identifies an important variable that could be empowered. In this sense, if low self-esteem levels could represent risk factors, higher levels of this variable could act as a possible protective factor.

Compulsive buying has high rates with other disorders including eating disorders [6,97,117,118,119]. Starting from these considerations, our aims were to test the comorbidity of these addictions, to verify the significant difference between compulsive and non-compulsive consumers regarding the effect of anxiety on the risk of developing eating disorders; and to test if in the group of compulsive consumers, the effect of anxiety on the risk of developing eating disorders is significantly stronger in all domains identified.

The results of the bivariate correlation showed a significant positive correlation between binge eating and compulsive buying and a significant negative correlation with self-esteem. Furthermore, there was also a significant positive correlation between anxiety and eating disorders and compulsive buying behavior.

Findings from the multi-group analysis support the differences in the effect of anxiety on eating disorders between compulsive and non-compulsive consumers. Due to the results of the multigroup invariance, it was also confirmed that, in the group of compulsive consumers, there was a significant and strong effect of trait anxiety on eating disorders in all domains identified (e.g., on Drive for thinness, on Bulimia, on Body Dissatisfaction, on Maturity Fears). These results confirmed data and findings reported in the current literature, where trait anxiety was identified as predictor of different types of addictions [97] and the hypotheses formulated (H3a,b) in this study.

## 8. Limitation of the Study and Future Directions

Despite the relevance, the present study had some limitations; the results of the research showed that the investigation of these behaviors should be further investigated, also by enrolling a larger sample. It is also necessary to exclude normal buying behavior and recurrent purchases are not necessarily evidence to support a diagnosis of compulsive buying behavior [6].

Another limitation of this study is the type of research design: being a cross-sectional study, longitudinal data is missing, and a true cause–effect relationship cannot be established; in addition, oversampling of certain characteristics such as the female sample. Therefore, it is intended to extend it by providing a multi-wave design to verify the cause–effect relationship. If replication studies support these findings and provide long-term follow-up, early recognition of specific traits (anxiety and self-esteem) could play an important predictive role for eating disorders and compulsive shopping, contributing to more effective preventive interventions [120].

Our findings also have some practical implications. Our research could help mental-health professionals (psychiatrists and psychologists) to better understand and determine which interventions, especially preventive ones, should be adopted, and to study their effectiveness and cost-effectiveness, considering the difficulty of finding resources for the proper functioning of health services [121,122,123,124]. Future research will have to evaluate the actual preventive interventions indicated, as these have been demonstrated to be effective and cost-effective in the prevention of other mental disorders [125]. According to our results, there is reason to think that identifying trait anxiety in advance, could be a resource to counteract the onset of compulsive behaviors and eating disorders; surely there will be improved approaches for prevention of, treatment of, and policies concerning behavioral addictions in the future.

## Figures and Tables

**Table 1:** Means, deviations and correlation values among variables.

	N	M	S.D.	α	1	2	3	4
1. EDI-2	352	297.07	51.64	0.92	1			
2. BES	352	25.7	7.9	0.89	0.49 **	1		
3. Self-esteem	352	2.97	0.58	0.79	−0.19 **	−0.30 **	1	
4. Trait Anxiety	352	48.98	6.96	0.71	0.45 **	0.14 *	−0.18 **	1
5. Compulsive Buying	352	1.54	0.74	0.68	0.34 **	0.20 **	−0.12	0.13 *

EDI-2 = Eating Disorder Inventory; BES = Binge Eating Scale; CB = Compulsive Buying; ** correlations are significant at the *p* < 0.001 level; * correlations are significant at the *p* < 0.05 level.

**Table 2:** Standardized indirect effects.

Predictor	Estimate	SE	BC 95% CI	
			LL	UL
Trait Anxiety → Self-esteem → EDI-2	−0.07 **	0.05	0.158	0.301
Trait Anxiety → Self-esteem → BES	−0.06 **	0.03	0.172	0.059
Trait Anxiety → Self-esteem → Compulsive Buying	−0.05 **	0.03	0.247	0.487

EDI-2 = Eating Disorder Inventory; BES = Binge Eating Scale; ** *p* < 0.01, SE = Standardized Estimates; BC = Bias Corrected; CI = Confidence Interval.

**Table 3:** Model Fit Indices, Models Comparison, and Summary of Invariance Tests Across Multigroup regarding compulsive buyer and not compulsive buyer.

	χ^2^	df	*p*	χ^2^/df	NFI	TLI	CFI	RMSEA [90% CI] *p*_close_	*p*_close_	AIC	BCC	ECVI	MECVI
Multigroup model for the total sample	596.581	132	<0.001	4.52	0.92	0.84	0.95	0.053 [0.049, 0.068]	1	812.581	888.823	0.646	0.654
Unconstrained model	662.003	168	<0.001	3.94	0.92	0.82	0.95	0.046 [0.042, 0.057]	1	806.047	856.875	0.799	0.805
Measurement model	675.804	174	<0.001	3.88	0.91	0.82	0.95	0.046 [0.042, 0.057]	1	807.752	854.345	0.811	0.815
Structural model	690.605	186	<0.001	3.71	0.92	0.82	0.95	0.046 [0.042, 0.057]	1	798.585	836.705	0.845	0.888
Groups/Models	∆χ^2 a^	∆*df* ^a^		*p* ^a^	∆CFI ^a^			∆χ^2 b^	∆*df* ^b^	*p* ^b^	∆CFI ^b^		
Measurement model	13.80	6		<0.001	<0.001								
Structural model	14.80	12		<0.001	<0.001			1	6	<0.001	<0.001		

Note. NFI = normed fit index; TLI = Tucker–Lewis index; CFI = comparative fit index; RMSEA = root mean square error of approximation; CI = confidence interval; AIC = Akaike information criterion; BCC = Brown-Cudeck criterion; ECVI = expected cross-validation index; MECVI = modified expected cross-validation index. ^a^ Assuming unconstrained model to be correct. ^b^ Assuming measurement model to be correct.

**Table 4:** Summary of path analysis between variables of our model among the compulsive/not-compulsive with equality constraints between the groups.

Paths of Interest			Not Compulsive Buyer		Compulsive Buyer	
			B (SE)	*p*	B (SE)	*p*
Trait Anxiety	→	Drive for thinness	−0.05	ns	−0.39	<0.001
Trait Anxiety	→	Bulimia	0.09	ns	0.36	<0.001
Trait Anxiety	→	Body Dissatisfaction	−0.01	ns	0.27	<0.05
Self Esteem	→	Interpersonal Distrust	−0.06	ns	−0.41	<0.001
Self Esteem	→	Interceptive Awareness	−0.09	ns	−0.25	<0.05
Trait Anxiety	→	Maturity Fears	0.04	ns	0.21	<0.05
Self Esteem	→	Asceticism	0.07	ns	−0.29	<0.05
Trait Anxiety	→	Impulse regulation	−0.15	<0.05	0.08	ns
Trait Anxiety	→	Ineffectiveness	0.17	<0.05	0.26	<0.05

B (SE) = standardized estimates; ns = not significant.

## Declarations

The authors developed their manuscript according to validated guidelines and manuals for their type of study (STROBE—Strengthening the reporting of observational studies in epidemiology) and to guidelines for auto evaluation of risk of bias in their research (JBCAT—Joanna Briggs critical appraisal tools; NHLBI SQAT—NHLBI Study Quality Assessment Tools).
